# Ninj2 regulates Schwann cells development by interfering laminin-integrin signaling

**DOI:** 10.7150/thno.76131

**Published:** 2022-10-17

**Authors:** Yuxia Sun, Xiang Chen, Cen Yue, Wenyi Yang, Shu Zhang, Zhimin Ou, Ying Chen

**Affiliations:** 1 State Key Laboratory of Cellular Stress Biology, School of Life Sciences, Xiamen University, Xiamen 361005, Fujian Province, China.; 2 Department of Neurology, The First Affiliated Hospital of Xiamen University, School of Medicine, Xiamen University, Xiamen, China.; 3 Fujian Key Laboratory of Brain Tumors Diagnosis and Precision Treatment, Xiamen Key Laboratory of Brain Center, Xiamen, China.; 4 Women and Children's Hospital, School of Medicine, Xiamen University, Xiamen, China.

**Keywords:** Ninj2, Peripheral Nervous System, Myelin, Schwann cell, ITGB1

## Abstract

**Rationale:** Myelin sheath is an important structure to maintain normal functions of the nerves. Nerve Injury-Induced Protein 2 (*Ninj2*) was found upregulated in Schwann cells (SC) upon injury. However, whether and how Ninj2 plays a role in myelination remain unknown.

**Methods:** In this study, we use transmission electron microscope imaging, immunofluorescent imaging, and behavioral tests to show the effects of Ninj2 on myelination and remyelination in peripheral nervous system (PNS) of SC-specific *Ninj2* knockout mice (*Dhh^cre/+^;Ninj2^fl/fl^*). For mechanism studies, we use RNA-Seq analysis to show the Ninj2-related pathways, and co-immunoprecipitation/mass-spectrometry to identify the Ninj2-interacting proteins in SCs. Furthermore, we evaluate the effect of integrin inhibitor GRGDSP during remyelination.

**Results:** Ninj2 negatively regulates SC development. *Ninj2*-deficient mice exhibit precocious myelination phenotype, as well as the accelerated remyelination process after sciatic nerve injury. Loss of *Ninj2* promotes myelination by promoting SC proliferation to augment its population. Mechanistically, Ninj2 interacted with ITGB1 on SC membrane, which inhibits laminin-integrin signaling. Removal of *Ninj2* induces the activity of laminin-integrin signaling, resulting in the improved myelination in the *Dhh^cre/+^;Ninj2^fl/fl^* mice. Inhibition of laminin-integrin signaling by integrin inhibitor GRGDSP sufficiently delays the remyelination process in the *Dhh^cre/+^;Ninj2^fl/fl^* mice with sciatic nerve injury.

**Conclusion:** Our study found Ninj2 as a negative regulator in the network controlling myelination in the PNS.

## Introduction

Schwann cells (SCs) form myelin sheath in the peripheral nerve system. They wrap axons to protect the rapid saltatory conduction of action potentials. Abnormal SC development and functional deficit usually lead to various kinds of peripheral neuropathies 1-3.

Development and myelination of SCs are controlled by a network constituted by multiple extracellular signals and intracellular factors. Among them, neuregulin 1 (NRG1) 4, 5, laminin-integrin signaling 6, 7 and G-protein coupled receptor 126 (Gpr126) 8 promote SC development, whereas Notch signaling and Sox2 play negative roles 9. The proper SC development requires precise balance of the positive and negative regulatory cues. During SC development, high mobility group (HMG)-type protein Sox10 10, Pit-Oct-Unc (POU)-homeodomain proteins Pou3f1 (Oct6) 11, zinc-finger proteins Krox20 (Egr2) 12 and Yin Yang 1 (YY1) 13 are the most important transcription factors promoting SC myelination. To date, new molecules are still being identified in the regulatory network for SC development.

Nerve injury-induced protein 2 (Ninj2), encoded by *Ninjurin 2* gene, belongs to the Ninjurin family. It was firstly identified as a surface adhesion molecule in SCs 14. Upon injury, the expression of *Ninj2* was upregulated in the SCs located in the distal nerve segment, inducing neurite outgrowth from dorsal root ganglion neurons through promoting hemophilic cellular interaction 14. Single nucleotide polymorphisms (SNPs) of *Ninj2* are reported to be correlated with increased risk of ischemic stroke incidence and multiple sclerosis (MS) 15-18. However, whether Ninj2 directly participates in the regulation of myelination and the underlying mechanism remain largely unknown.

Here, using SC-specific *Ninj2* mutant mice, we found that Ninj2 inhibited SC development during either physiological myelination or remyelination after injury in sciatic nerves. Our data thus identify Ninj2 as a novel negative regulator in the regulation of myelination, which might contribute to fulfilling the regulatory network for SC development.

## Results

### Loss of *Ninj2* promotes precocious myelination in peripheral nerve system

To examine the expression of *Ninj2* in the peripheral nerve system, we used co-immunostaining imaging for Ninj2 and the SC lineage marker Sox10 in the sciatic nerves. As shown in Figure S1A, *Ninj2* was substantially expressed in Sox10^+^ SCs. During the nerve development, the expression of *Ninj2* increased at the onset of myelination from postnatal day 0 (P0) to 14 (P14), and then declined before adulthood (Figure S1B).

**Figure 1:**
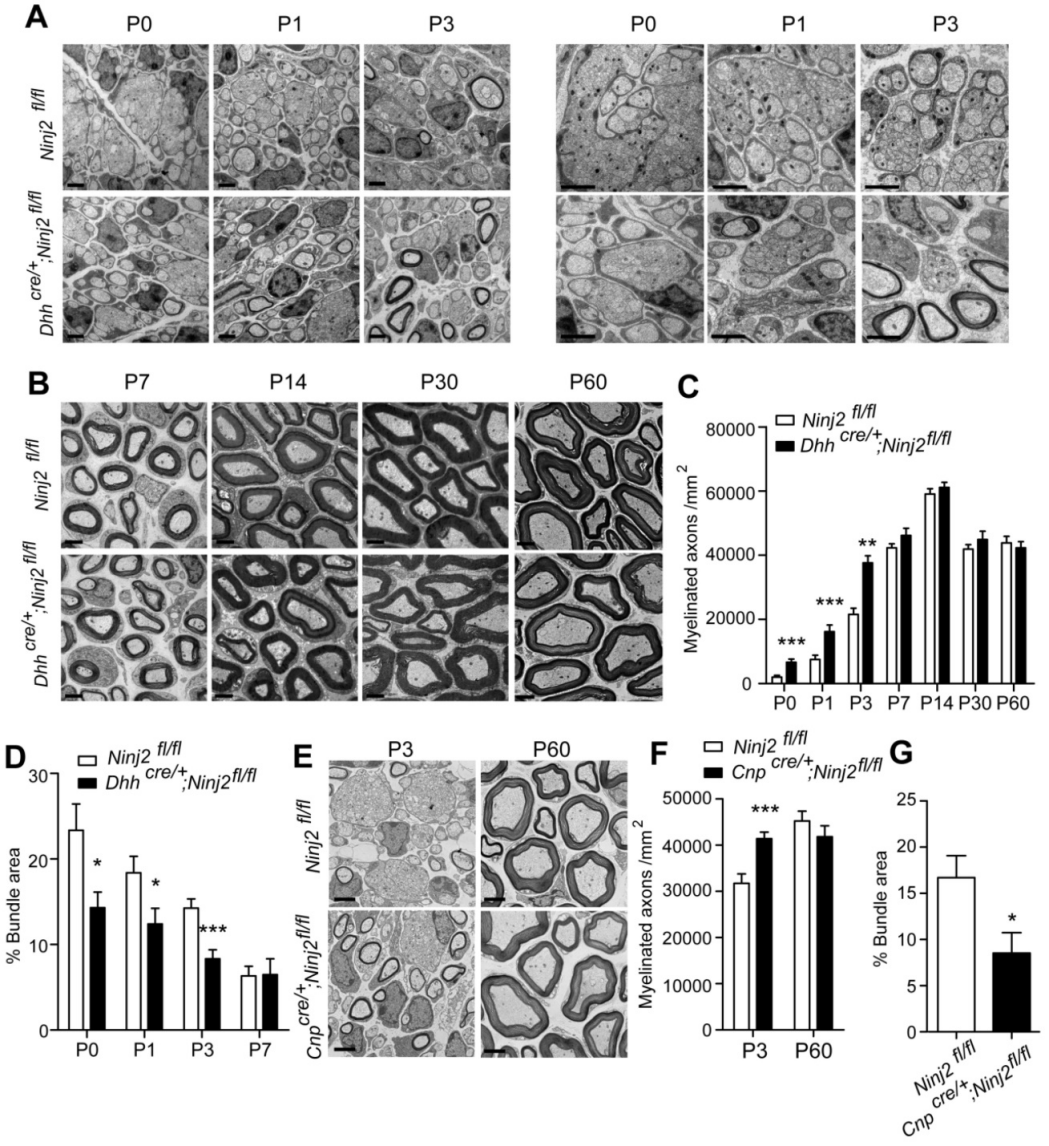
** Loss of *Ninj2* in SCs promotes precocious myelination. (A-B)** Electron microscopic examination of the sciatic nerve from WT or *Dhh^cre/+^;Ninj2^fl/fl^* mice from postnatal day 0 to 60 as indicated. Scale bar, 2 µm. **(C-D)** The myelinated axon numbers and the percentages of bundle area were quantified. **(E-G)** Electron microscopic examination of the sciatic nerve from WT or *Cnp^cre/+^;Ninj2^fl/fl^* mice at P3 and P60. Scale bar, 2 µm (E). The myelinated axon numbers (F) and the percentages of bundle area were quantified (G). For all panels, data were obtained from at least 6 mice. *P < 0.05, **P < 0.01, ***P < 0.001, students' t test.

To study the role of Ninj2 in SC development, we generated a conditional *Ninj2* knockout mice by removing the exon 2 of the *Ninj2* allele through a recombination mediated by a SC-specific cre line, Desert-hedgehog (Dhh)-cre 11 (Figure S2). In the *Dhh^cre/+^;Ninj2^fl/fl^* mice, we found that loss of *Ninj2* promoted precocious myelination in sciatic nerves. Using transmission electron microscope imaging, we clearly observed an early onset of radial sorting in the sciatic nerves of the *Dhh^cre/+^;Ninj2^fl/fl^* mice. The *Dhh^cre/+^;Ninj2^fl/fl^* mice had higher numbers of myelinated axons and smaller areas of bundles at P0 to P3 (Figure 1A-D), without change on G-ratio (Figure S3), compared with the WT mice. Similar precocious myelination phenotype was obtained in the *Cnp^cre/+^;Ninj2^fl/fl^* mice at P3, another mice strain with *Ninj2* knockout in glial cells (Figure 1E-G) 19. Myelinogenesis then became comparable at P7 and persisted to adulthood between WT and mutant mice. After P7, either *Dhh^cre/+^;Ninj2^fl/fl^* or *Cnp^cre/+^;Ninj2^fl/fl^* mice had morphology of the myelin sheath as the WT mice (Figure 1B and 1E). Furthermore, we performed CatWalk gait analysis and electrophysiological recordings of compound muscle action potentials (CMAPs) and found that WT and *Dhh^cre/+^;Ninj2^fl/fl^* mice had similar paw area (Figure S4A), as well as nerve conduction velocity and mean peak of CMAP amplitude (Figure S4B), supporting the fact that WT and *Dhh^cre/+^;Ninj2^fl/fl^* mice had similar level of myelinogenesis at adulthood.

### Ablation of *Ninj2* promotes SC proliferation

**Figure 2:**
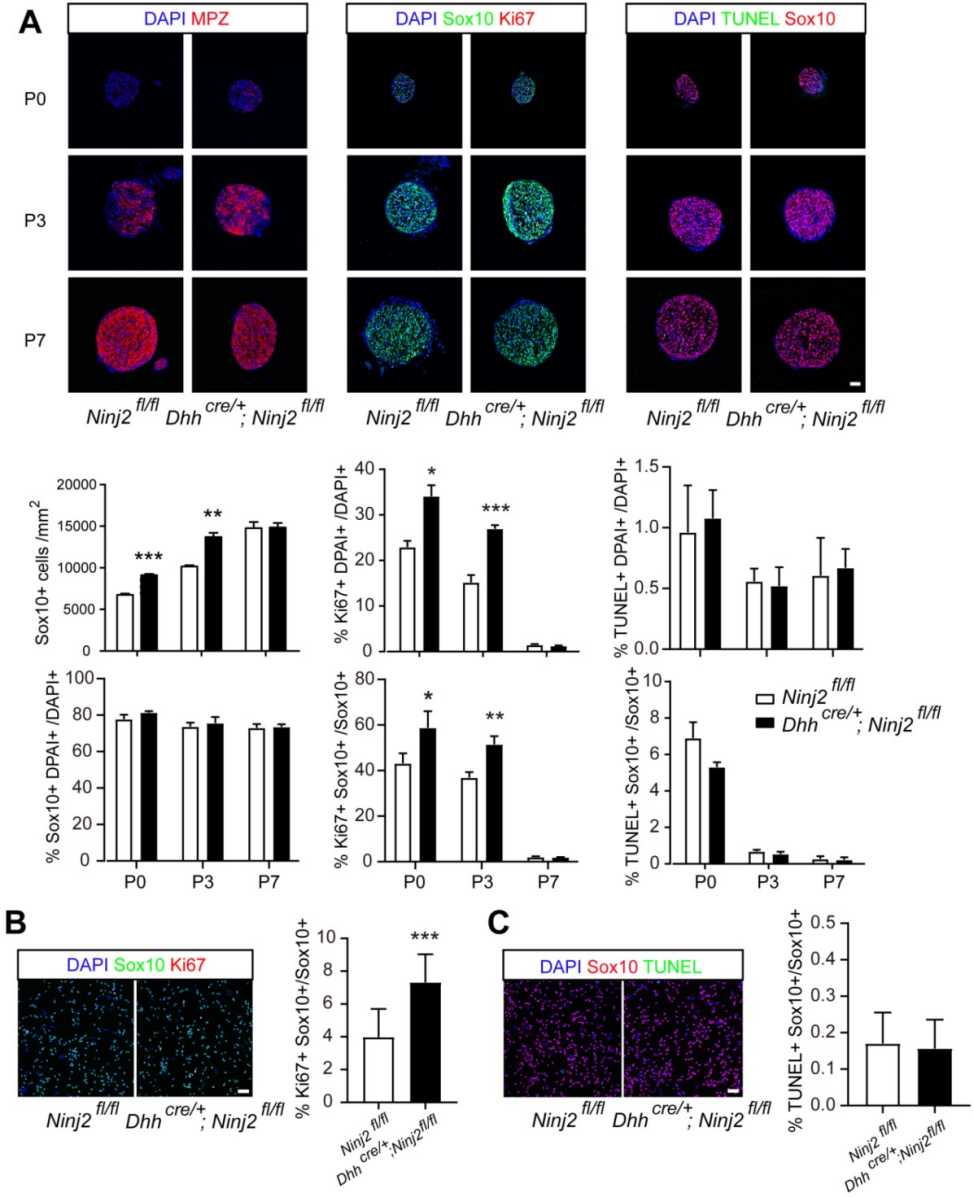
** Loss of *Ninj2* promotes SC proliferation and increased Sox10^+^ cell number. (A)** Immunofluorescent staining against Sox10, Mpz, Ki67, and TUNEL were performed in the sciatic nerve sections from WT or *Dhh^cre/+^;Ninj2^fl/fl^* mice at P0, P3, and P7, Scale bar, 50 µm. The Sox10^+^ cell density, the proportions of Sox10^+^, Ki67^+^ and TUNEL^+^ cells in total cell population, and the proportions of Ki67^+^Sox10^+^, TUNEL^+^Sox10^+^ cells in total Sox10^+^ cells were quantified and shown at the lower panels, respectively. **(B-C)** Immunofluorescent staining against Sox10, Ki67, and TUNEL were performed in primary cultured SCs isolated from WT or *Dhh^cre/+^;Ninj2^fl/fl^* mice. Scale bar, 50 µm. For all panels, data were obtained from at least 6 mice or 3 independent experiments, *P < 0.05, **P < 0.01, ***P < 0.001, students' t test.

To find out the reason that loss of *Ninj2* promoted precocious myelination in mice, we performed experiments to study the effects of Ninj2 during SCs development. Immunofluorescent imaging displayed that loss of *Ninj2* enlarged Sox10^+^ SC population at P0 to P3, resulting in the increased number of mature SCs (Mpz^+^) (Figure 2A). Furthermore, we discovered that *Ninj2* knockout promoted SC proliferation, but had no effect on cell survival, as indicated by the proportions Ki67^+^Sox10^+^, and TUNEL^+^Sox10^+^ cells in total Sox10^+^ cells (Figure 2A). In cultured primary SCs, similar results were observed (Figure 2B-C). These data suggested that loss of *Ninj2* in SCs increased Sox10^+^ cell proliferation, hence leading to enlarged SC population and precocious myelination in sciatic nerves.

### Loss of *Ninj2* accelerates remyelination in peripheral nerve system

**Figure 3:**
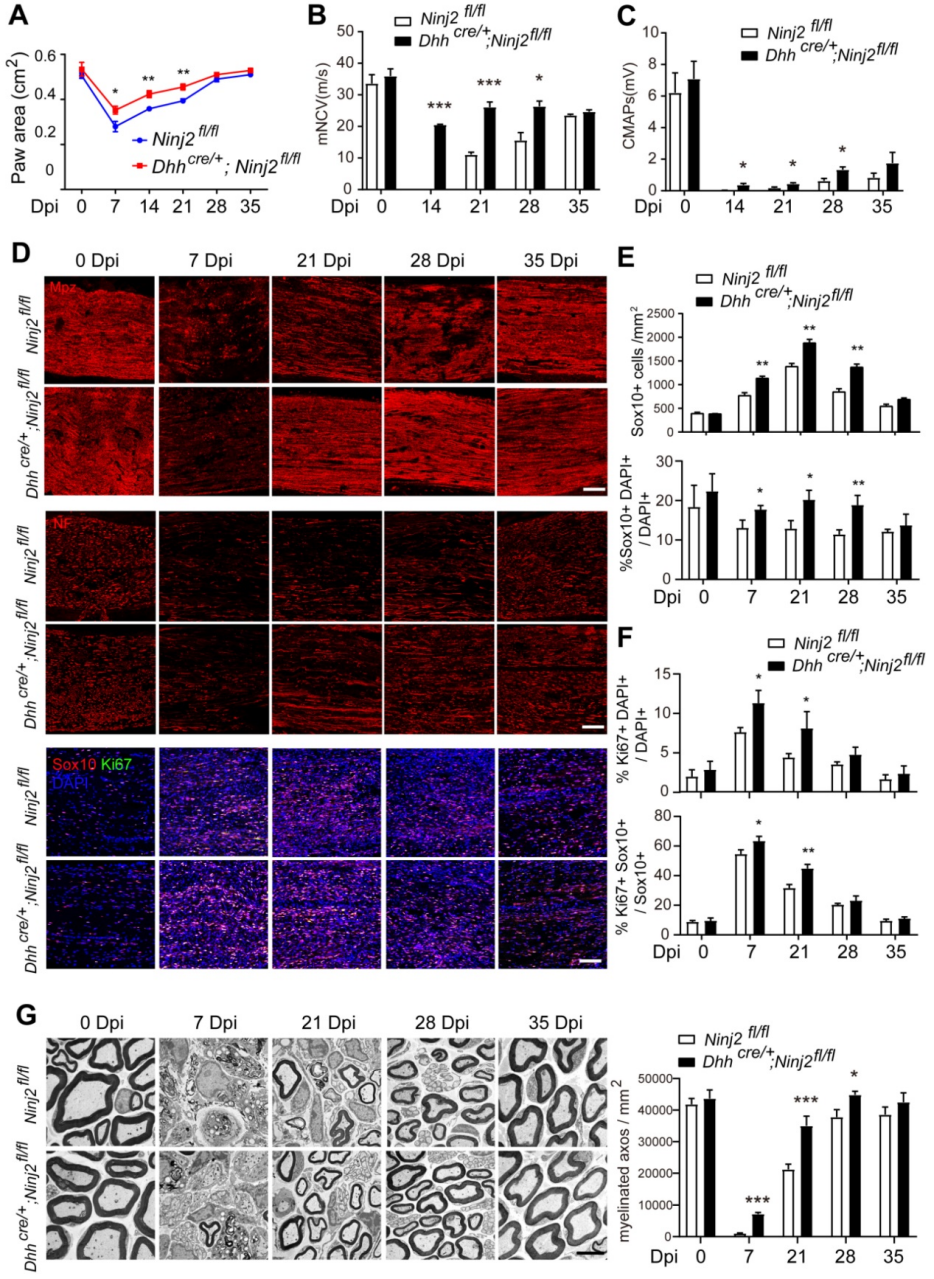
** Ablation of *Ninj2* in SCs accelerates sciatic nerve remyelination after injury. (A)** Paw area in DigiGait analysis with the WT or *Dhh^cre/+^;Ninj2^fl/fl^* mice at indicated time points after injury. **(B-C)** Electrophysiological recording of CMAPs with the WT or *Dhh^cre/+^;Ninj2^fl/fl^* mice at indicated time points after injury. **(D)** Immunofluorescent staining against Mpz, Neurofilament (NF), Sox10 and Ki67 in the sciatic nerve sections at 0-35 dpi from WT or *Dhh^cre/+^;Ninj2^fl/fl^* mice at the age of P60 that received crush injury. Scale bar, 15 µm. **(E and F)** The Sox10^+^ cell density, the proportions of Sox10^+^ and Ki67^+^ cells in total cell population, and the proportion of Ki67^+^Sox10^+^ cells in total Sox10^+^ cells were quantified. **(G)** Electron microscopy analysis was performed with the sciatic nerve samples at 0-35 dpi from WT or *Dhh^cre/+^;Ninj2^fl/fl^* mice at the age of P60 that received crush injury, Scale bar, 5 µm. The myelinated axons numbers at the indicated time points were calculated and shown in the right panel. For all panels, data were obtained from at least 6 mice. *P < 0.05, **P < 0.01, ***P < 0.001, students' t test.

We then tested whether loss of *Ninj2* in SCs promoted remyelination. Both WT and *Dhh^cre/+^;Ninj2^fl/fl^* mice received a crush injury on their sciatic nerves to introduce myelin injury. We performed CatWalk gait analysis and electrophysiological recordings to physiologically evaluate their recovery status. Figure 3A indicated that the *Dhh^cre/+^;Ninj2^fl/fl^* mice had improved motor coordination and locomotion than the WT mice at 7, 14 and 21 dpi, which was highly consistent with their myelin recovery processes. The *Dhh^cre/+^;Ninj2^fl/fl^* mice also showed improved nerve conduction velocity and mean peak of CMAP amplitude during the remyelination process than their WT counterparts (Figure 3B-C).

The sciatic nerve samples were then collected at 0, 7, 21, 28 and 35 days post injury (dpi) for immunofluorescent imaging targeting Mpz, Neurofilament, Ki67 and Sox10 (Figure 3D-F). At 7 dpi, the Mpz^+^ area were dramatically decreased in the injured area in the sciatic nerves from both WT and *Dhh^cre/+^;Ninj2^fl/fl^* mice. However, ablation of *Ninj2* in SCs significantly accelerated the remyelination process. At 21 and 28 dpi, a larger Mpz^+^ area could be observed in the *Dhh^cre/+^;Ninj2^fl/fl^* mice than their WT counterparts. The density of Neurofilament indicated improved neurite outgrowth in the *Ninj2*-deficent mice, which was highly consistent with their remyelination process. The accelerated remyelination in *Dhh^cre/+^;Ninj2^fl/fl^* mice was tightly connected to the augmented Sox10^+^ and Ki67^+^SCs in the injury area. As shown, larger numbers of Sox10^+^cells, and a higher proportion of Ki67^+^Sox10^+^cells in total Sox10^+^cells could be seen in the injured sciatic nerves from *Dhh^cre/+^;Ninj2^fl/fl^* mice at 7, 21 and 28 dpi, compared to that in WT mice (Figure 3D-F).

We have also examined the remyelination status at these time points by electron microscopy analysis. As shown in Figure 3G, the *Ninj2*-deficient mice showed accelerated remyelination process, which was highly consistent with what we observed through immunofluorescent staining. Taken all of the results together, we could conclude that loss of *Ninj2* promoted remyelination.

### Ninj2 inhibits laminin-integrin signaling by an interaction with ITGB1 in SCs

**Figure 4:**
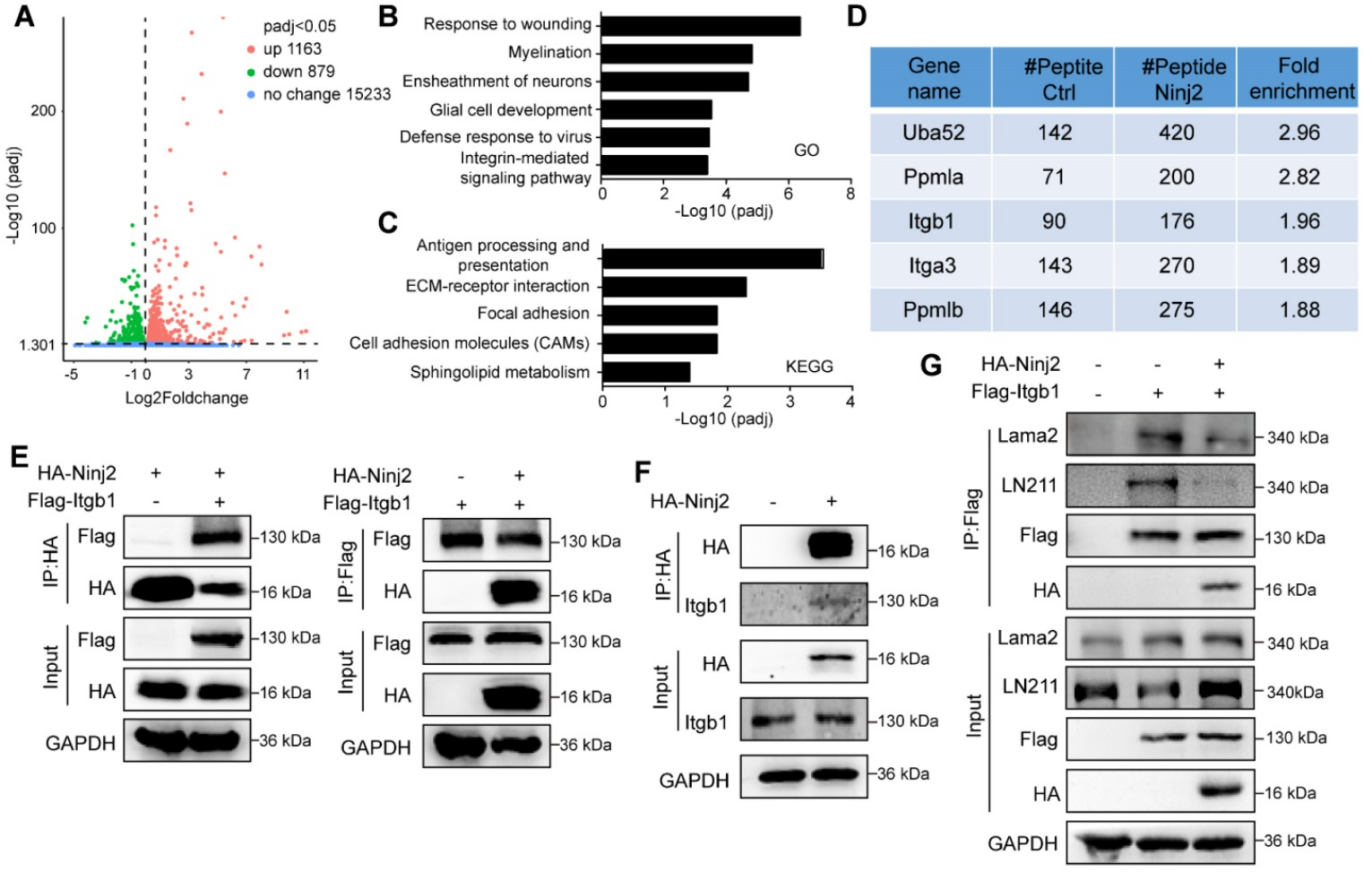
** Ninj2 regulates SC development through antagonizing laminin-integrin signaling. (A)** RNA-Seq analysis with total mRNAs that isolated from rat SCs transduced with scramble/Ninj2 shRNA. **(B-C)** Gene ontology (B) and KEGG analysis (C) of genes whose expression levels were significantly changed. **(D)** The Ninj2-interacting proteins by mass-spectrum analysis on WT or Ninj2-overexpressing S16 cells. **(E-F)** Co-IP assays were performed to validate Ninj2 and ITGB1 interaction in HEK293T cells (E) and rat SCs (F). **(G)** S16 cells were transduced with control/Itgb1-overexpressing vector alone, or in combination of control/Ninj2-overexpressing vectors as indicated, and then harvested for Co-IP assays to examine the interaction between ITGB1 and Lama2. All co-IP data were observed in at least 3 independent experiments.

To demonstrate the mechanism underpinning the inhibitory effect of Ninj2 in SCs development, we performed RNA-Seq analysis using WT and Ninj2 knockdown SCs. Knockdown of *Ninj2* induced the expression levels of 1163 genes, and reduced the expressions of 879 genes (Figure 4A). Gene ontology analysis indicated that those genes whose expression levels were significantly changed involved in the biological processes including response to wounding, myelination, ensheathment of neurons, glial cell development, defense response to virus, and integrin-mediated signaling pathway (Figure 4B). Further, according to the KEGG database, these genes were categorized into signaling pathways such as antigen processing and presentation, ECM-receptor interaction, focal adhesion, cell adhesion molecules (CAMs), and sphingolipid metabolism (Figure 4C). Both GO and KEGG analysis pointed out that loss of Ninj2 substantially affected the signaling pathways that controlled by the extracellular matrix (ECM) components, such as laminin and integrin. ECM components, especially laminins and integrins, played vital roles in radial sorting by controlling SC proliferation, polarization and formation of cellular protrusion 20-22. We thus hypothesized that loss of* Ninj2* induced SC development through laminin-integrin mediated signaling.

To test our hypothesis, we performed co-immunoprecipitation assay (Co-IP) using FLAG antibody in WT or Ninj2-overexpressing S16 cells, and then screened the Ninj2-interacting proteins through mass-spectrometry analysis. Upon screening, we discovered that integrin b1 (ITGB1) was a potential Ninj2 interacting protein (Figure 4D). Co-IP assays were then performed to validate such interaction. As shown, Ninj2 bound to ITGB1 in both HEK293T cells and SCs (Figures 4E and F). The interaction between Ninj2 and ITGB1 seemed to competed out the recruitment of laminin onto ITGB1. In S16 cells, the interaction between ITGB1 and the endogenous laminin 211 (LN211) could be detected, when *Ninj2* was overexpressed in the cells, the ITGB1/Lama2 interaction was greatly decreased (Figure 4G). Therefore, we believed that Ninj2 served as a competitive inhibitor of laminin for integrin, which thus interrupting the laminin-integrin signaling, eventually resulted in its repressive effects in SC development.

### Loss of *Ninj2* activates Focal adhesion kinase (FAK) signaling in SCs

**Figure 5:**
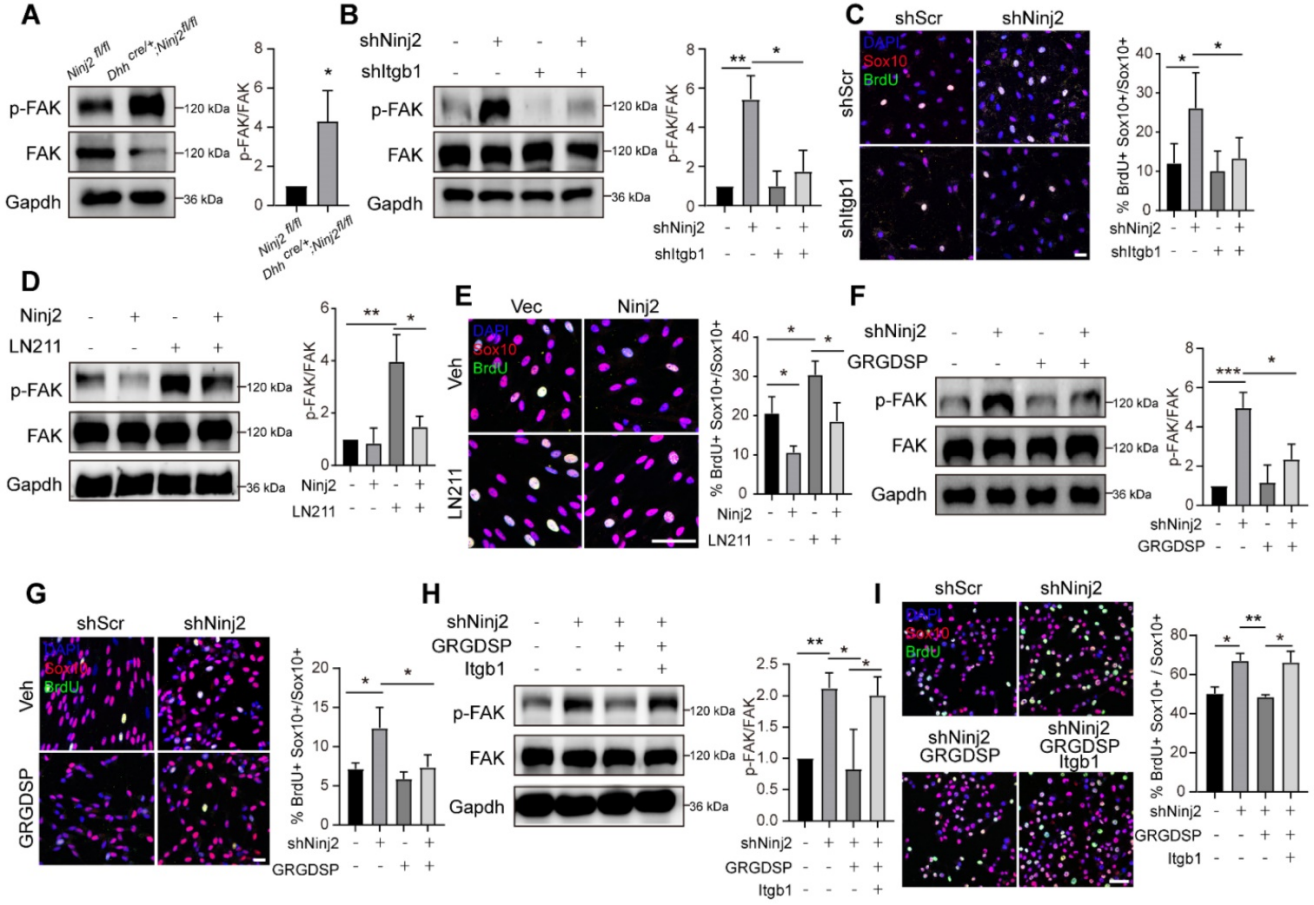
** Ninj2 competes with ITGB1 in the laminin-integrin signaling. (A)** Western blot analysis against phosphorylated FAK in sciatic nerves of WT or *Dhh^cre/+^;Ninj2^fl/fl^* mice. **(B-C)** Western blot analysis against phosphorylated FAK (B), and immunofluorescent staining against Sox10 and BrdU (C) in rat SCs transduced with scramble shRNA, shNinj2, shItgb1 or a combination of shNinj2 and shItgb1, as indicated. **(D-E)** Western blot analysis against phosphorylated FAK (D), and immunofluorescent staining against Sox10 and BrdU (E) in rat SCs transduced with control/Ninj2-overexpressing vector, and treated vehicle or 1 µg/mL LN211 for 48 h. **(F-G)** Western blot analysis against phosphorylated FAK (F), and immunofluorescent staining against Sox10 and BrdU (G) in rat SCs transduced with scramble/Ninj2 shRNA, and with or without (10 µM) GRGDSP treatment for 48 h. **(H-I)** Western blot analysis against phosphorylated FAK (H), and immunofluorescent staining against Sox10 and BrdU (I) in S16 cells transduced with scramble/Ninj2 shRNA, control/Itgb1-overexpressing vector, and with or without (10 µM) GRGDSP treatment for 48 h, as indicated. For all panels, data were obtained from at least 3 independent experiments. Scale bar, 50 µm. For all panels, *P < 0.05, **P < 0.01, ***P < 0.001, Two-way ANOVA test.

Since Ninj2 took part in the interaction of laminin-integrin, we then explored the effects of Ninj2 on FAK signaling, a major downstream pathway of laminin-integrin controlling SC proliferation 23, 24. We found that knockdown of *Ninj2* increased the phosphorylated FAK level in sciatic nerves (Figure 5A). When *Itgb1* was further knocked down, the activation of FAK signaling was largely abolished (Figure 5B). The level of SC proliferation was consistent with the activation of the FAK signaling. Single *Ninj2* knockdown increased BrdU^+^ SC number. But *Ninj2* and *Itgb1* double knockdown SCs showed similar level of proliferation as WT SCs (Figure 5C). In contrast, overexpression of *Ninj2* inhibited the activation of FAK signaling by Laminin 211 (LN211) administration, and attenuated the inductive effects of LN211 on cell proliferation (Figure 5D-E). Furthermore, we used GRGDSP, an established integrin signaling inhibitor 25, to strengthen the significance of laminin-integrin signaling in the control of SC proliferation by Ninj2. Similar with the effects of Itgb1 knockdown, GRGDSP treatment abolished, at least partially, the activation of FAK and induction of SC proliferation by knockdown of *Ninj2* (Figure 5F-G). Furthermore, the effects of GRGDSP in the *Ninj2*-deficient cells were largely abolished by overexpression of Itgb1 (Figure 5H-I). Collectively, we could conclude that laminin-integrin-FAK signaling was the pathway mediating the effects of Ninj2 in SC development.

### Interrupting laminin-integrin signaling delays remyelination in *Dhh^cre/+^;Ninj2^fl/fl^* mice

**Figure 6:**
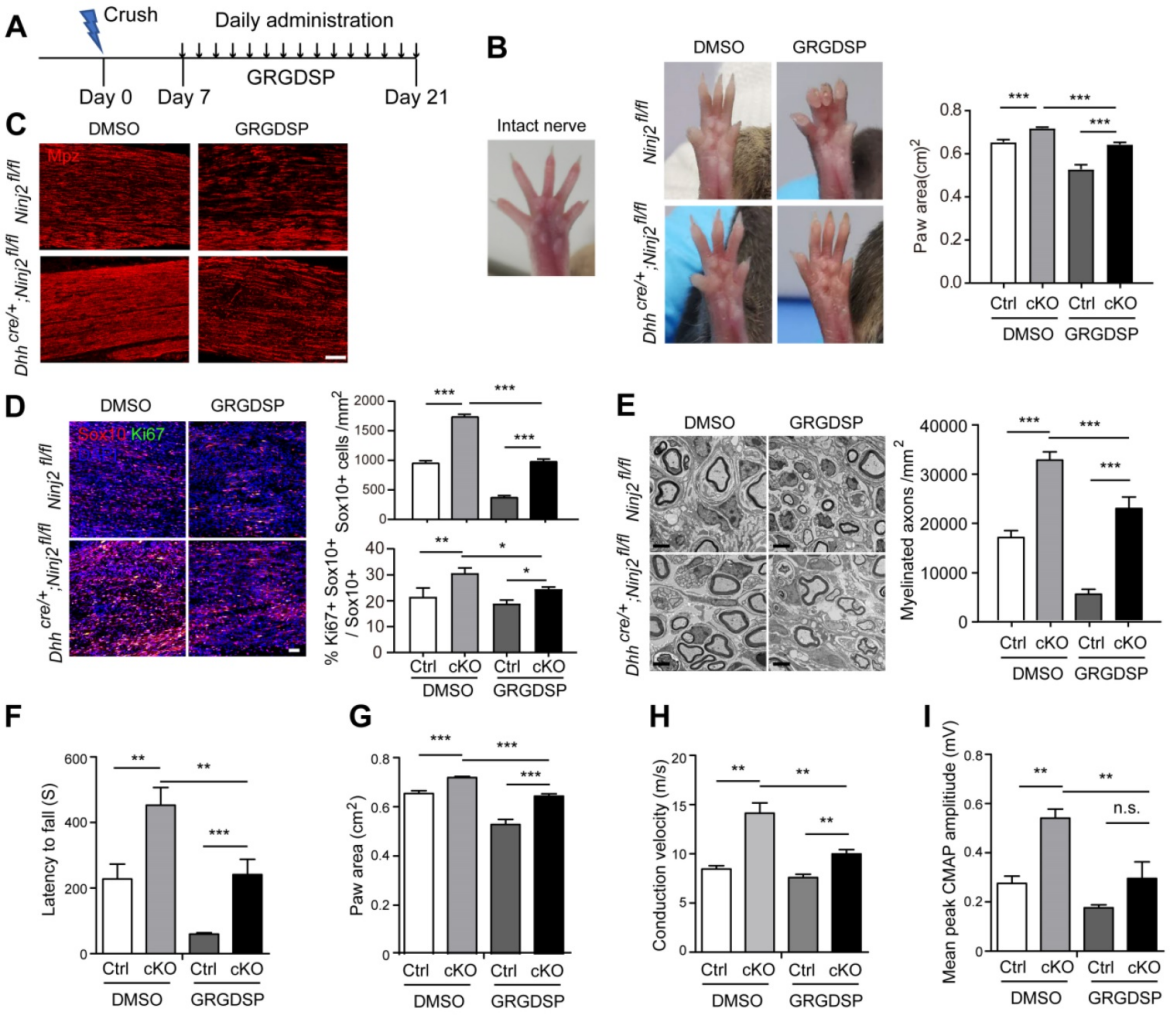
** Inhibition of integrin signaling attenuates the rapid remyelination characteristics in *Dhh^cre/+^;Ninj2^fl/fl^* mice. (A)** The diagram showing the strategy of the sciatic nerve injury and GRGDSP administration model. The following experiments were performed at the end of the experiment. **(B)** Photographs were collected to show the toe-spreading of the mice. And paw areas were quantified. **(C-D)** Immunofluorescent staining against Mpz (C), Sox10 and Ki67 (D) were performed in the sciatic nerve sections, the density of the Sox10^+^ cells, and the proportion of Ki67^+^Sox10^+^ cells in Sox10^+^ cells were quantified and shown on the right panel of D, Scale bar, 15 µm (C) and 50 µm (D). **(E)** Electron microscopic examination of the sciatic nerve were performed, scale bar, 2 µm, and myelinated axon numbers were quantified. **(F-I)** Rotarod test (F), DigiGait analysis (G) and Electrophysiological recording of CMAPs (H-I) were performed. For all panels, data were obtained from at least 6 mice. *P < 0.05, **P < 0.01, ***P < 0.001, Two-way ANOVA test.

As we found that integrin signaling pathway mediated the regulatory effect of Ninj2 on SC development, we then went on to test whether inhibition on integrin activity could attenuate the accelerated remyelination process by *Ninj2* knockout *in vivo*. WT or *Dhh^cre/+^;Ninj2^fl/fl^* mice that received a crush injury on their sciatic nerves were then administrated with vehicle or 1mg/kg of GRGDSP at the injured area from 7 to 21 dpi (Figure 6A). At 21 dpi, compared with the untreated mice, both GRGDSP-treated WT and *Dhh^cre/+^;Ninj2^fl/fl^* mice showed reduced toe-spreading reflex (Figure 6B), suggesting that their remyelination process was greatly delayed. Immunofluorescent staining was then performed to evaluate the remyelination status of these mice. As indicated by the Mpz, Ki67 and Sox10 staining, GRGDSP treatment apparently reduced the densities of Mpz^+^, Ki67^+^ and Sox10^+^ SCs in the injured area in the *Dhh^cre/+^;Ninj2^fl/fl^* mice (Figure 6C-D). EM imaging further indicated that GRGDSP treatment reduced myelinated axon numbers in *Dhh^cre/+^;Ninj2^fl/fl^* mice at 21 dpi (Figure 6E). Moreover, GRGDSP-treated WT and *Dhh^cre/+^;Ninj2^fl/fl^* mice showed reduced motor coordination and locomotion, and nerve conduction velocity and mean peak of CMAP amplitude (Figure 6F-I). All of these data evidenced that inhibition of integrin signaling sufficiently attenuate the rapid remyelination characteristics in *Dhh^cre/+^;Ninj2^fl/fl^* mice, robustly supporting the essential role of integrin signaling in the control of SC development by Ninj2.

## Discussion

In our current study, we identify Ninj2 is a novel inhibitory factor that negatively regulates myelin sheath development in peripheral nerve system. Loss of *Ninj2* in SCs promotes precocious myelination and accelerates remyelination after myelin injury in sciatic nerves by inducing SC proliferation and enlarging their population. Mechanistically, Ninj2 interacts with ITGB1 on the SC membrane, and it interrupts the binding of LN211 onto ITGB1, which leads to an inhibitory effect on SC development.

Ninj2 was firstly reported as a homophilic cellular adhesion molecule, it was upregulated in SCs surrounding the distal segment of injured nerve 14. However, whether and how Ninj2 is involved in myelin development remained largely unknown. Until recently, we found that loss of *Ninj2* in oligodendrocytes inhibited oligodendrocyte and myelin development, and then induced depressive-like behaviors. Herein, in the sciatic nerves, we observed a totally different effect of Ninj2 knockdown on myelination.

To explore the molecular basis underlying such phenotype. We firstly performed co-IP and mass-spectrometry analysis to find Ninj2 interacting proteins, and RNA-Seq analysis to show the pathways in response to *Ninj2* deletion. According to the data, Ninj2-interacting proteins included ITGB1, one of the most common receptors for the ECMs. RNA-Seq analysis consistently pointed out that the differentially expressed genes upon Ninj2 deletion were involved in integrin-mediated signaling pathways or ECM-receptor interaction. ECM signaling pathway is one of the most important machinery that controlling SC development. It has been reported to regulate SC proliferation, polarization and formation of cellular protrusion 20-23. In our current study, we observed a substantial interaction of Ninj2 and ITGB1, and loss of Ninj2 activated the downstream FAK signaling of LN211/ITGB1. On the other hand, overexpression of Ninj2 effectively abolished the activation of FAK signaling by LN211 treatment. These results indicated that Ninj2 played a role in the laminin-integrin-FAK regulatory axis.

Furthermore, to establish that Ninj2 majorly functioned through the laminin/integrin signaling, we interrupted this signaling pathway in the Ninj2 deficient SCs by administration of GRGDSP, an inhibitor against integrin. As expected, GRGDSP efficiently blocked the activation of FAK, a downstream pathway of laminin/integrin, upon *Ninj2* deletion. These effects were highly consistent with that caused by knockdown of Itgb1. In fact, GRGDSP was reported as a pan inhibitor for integrins. Although we could not obtain specific inhibitory effect against ITGB1 by GRGDSP administration, when we overexpressed ITGB1 in the Ninj2-deficient cells treated with GRGDSP, the inhibitory effects of GRGDSP on FAK signaling activation and cell proliferation were greatly rescued. Therefore, we could, at least partially, proved our hypothesis that integrin signaling pathway mediated the effects of Ninj2 in the SCs.

Interestingly, as we recently reported, Ninj2 showed distinguished functions in the central nervous system. In oligodendrocytes, loss of *Ninj2* induced necroptotic programmed cell death, leading to delayed oligodendrocyte development and myelin defects. When we explored the molecular basis, we found that Ninj2 competitively inhibited the interaction of TNFα and TNFR1 by its interaction with TNFR1, and thus blocked the activation of TNF signaling. Loss of *Ninj2* sensitized oligodendrocytes to TNFα induced necroptosis. However, in SCs, we did not see an induction of cell death after Ninj2 deletion in SCs. Co-IP and mass-spectrometry analysis did not show a significant differential binding pattern between Ninj2 and TNFR1, either. Instead, ITGB1 was one of the proteins that most significantly interacted with Ninj2 in SCs. We then proved that ITGB1 and its downstream FAK signaling majorly mediated the effects of Ninj2 in SCs.

Notably, although we observed that Ninj2 predominantly interacted with ITGB1 in SCs, and with TNFR1 in oligodendrocytes, resulting at diverse outcomes for myelination, the functions of laminin/integrin or TNFα/TNFR1 were not specific in these two cells. As reported, integrins and its downstream pathways regulated oligodendrocyte development. On the other hand, TNFR1 may also played a role in the control of SC development. The underpinning mechanism of the differential preference of Ninj2 interacting proteins in SCs and oligodendrocytes remained an open question. Further studies are in great need to demonstrate the details in these regulation processes.

In summary, our current study identifies Ninj2 as an inhibitor for myelination in the peripheral nerve system. Although ligands or inhibitors of Ninj2 are still need to be fully developed, it could be considered as a therapeutic target for demyelinating diseases.

## Methods

### Animals

The *Ninj2^fl/fl^* mice were generated by Shanghai Biomodel Organism Science and Technology Development Company. *Dhh^cre/+^* and *Cnp^Cre/+^* mice were kindly provided by Dr. Q. Richard Lu (Cincinnati Children's Hospital Medical Center). Sprague-Dawley rats were purchased from Xiamen University Laboratory Animal Center. All mice were maintained in the Xiamen University Laboratory Animal Center. Animals of both sexes were used in the study, and littermates were used as controls.

All of the animal experiments were approved by and performed according to the experimental guidelines of the Animal Care and Use Committee of Xiamen University.

### Antibodies

The antibodies used in immunofluorescence and western blot included Ninj2 (R&D systems, Cat# AF5056), Cleaved-Caspase 3 (Cell Signaling Technology, Cat# 9661), phosphorylated MLKL (abcam, Cat# ab196436), Laminin 211 (abcam, Cat# ab7463), Lama2 (Boster, Cat# BA3201-2), Sox10 (abcam, Cat# ab155279), Sox10 (abcam, Cat# ab216020), Mpz (abcam, Cat# ab31851), Neurofilament (abcam, Cat# ab8135), Ki67 (Gene Tex, Cat# GTX16667), phosphorylated FAK (abcam, Cat# ab4792), GAPDH (Proteintech, Cat# 60004-1-lg), HA (Santa Cruz, Cat# sc-7392), and FLAG (sigma, Cat# B3111).

### Immunofluorescence

Mice were perfused with 4% paraformaldehyde (PFA), and prepared with 16 μm frozen sections. The sections were incubated in blocking solution for 30 min at room temperature. Primary antibodies were then applied overnight at 4 °C. The next day, sections were incubated with the secondary antibodies for 2 h at room temperature.

### TUNEL

Immunostaining against TUNEL were performed using DeadEndTM Fluorometric TUNEL System (Promega, Cat# G3250) following manufacture's protocol.

### Co-immunoprecipitation

Cells were transfected with plasmid for 48 h, then lysed and incubated with anti-HA or FLAG antibodies overnight at 4 °C. The complexes were precipitated with 10 μL of Protein A Magnetic Beads (MCE, Cat# HY-K0203) with gentle agitation at 4 °C for 2 h. The immunoprecipitated protein complexes were subjected to subsequent experiments.

### Mass spectrometry

A stable *Ninj2*-overexpressing S16 cells were lysed and incubated with Anti-FLAG® M2 Magnetic Beads (Sigma, Cat# M8823) for 4 h at 4 °C. The complexes were eluted by 3X FLAG® Peptide (Sigma, Cat# F4799), and precipitated with acetone. The purified protein complexes were isolated by a SDS-PAGE gel electrophoresis and processed for proteomic mass spectrometry analysis.

### Primary rat Schwann cells culture

Sciatic nerves were isolated from P0-P1 rats, and digested with 1 mg/ml collagenase A (Sigma). Schwann cells were plated into poly-D-lysine (Sigma) coated dish and grown in DMEM High Glucose (Hyclone) supplemented with 10% fetal bovine serum (Hyclone) and 1 mM Ara-C (Sigma) for 3 days. After passage, Schwann cells were cultured with proliferation medium (DMEM High Glucose supplemented with 10% fetal bovine serum, 10 ng/ml NRG1 (R&D Systems), 5 μM forskolin (Sigma), 1% L-glutamine (Hyclone, Cat# SH30034) and 1% penicillin/streptomycin). To differentiate, Schwann cells were cultured in differentiation medium (DMEM High Glucose supplemented with 0.5% fetal bovine serum and 1 mM dibutyl cyclic AMP (Sigma) with 1% L-glutamine and 1% penicillin/streptomycin).

### RNA-Seq analysis

RNA-Seq analysis with rat Schwann cells transfected with scramble or Ninj2- shRNA were performed by Novogene (Beijing, China). All RNA-Seq data were aligned to Rn5 by Hisat2 v2.0.5. FeatureCounts v1.5.0-p3 and StringTie (v1.3.3b) were used to generate gene counts. Differentially expressed genes were identified using DESeq2 with fold change >1.5 and p < 0.05.

### Electron microscopy

Sciatic nerves were dissected and fixed immediately in a fixative solution (2.5% glutaraldehyde and 0.01 M phosphate buffer, pH = 7.4) at room temperature for 2 h, and then at 4 °C overnight. The next day samples washed in phosphate buffer, postfixed in 1% osmium tetroxide, dehydrated in graded ethanol series, and embedded in epoxyresin (Durcupan). The ultrathin sections were investigated under a Transmission Electron Microscope (TEM) (Hitachi HT-7800).

### Electrophysiology

Electrophysiological measurements were performed as previously described 26. Compound muscle action potentials (CMAPs) were recorded with an ADinstruments PowerLab 26T instrument. Nerve conduction velocities were calculated from the distance between proximal and distal stimulation electrodes and the latency difference between the CMAPs after successive proximal and distal stimulation. CMAP amplitudes were calculated peak to peak.

### Rotarod test

Each mouse was allowed to acclimate to the rotarod apparatus (Ugo Baslie 47650) at 10 rpm and 20 rpm for 5 min and trained 3 trials daily for 2 consecutive days prior to the first test. In the test, mice were subsequently placed on an accelerating rotarod that accelerated from 5 to 35 rotations per minute over a period of 2 min. Each test was composed of 5 trials on the rotarod with a rest period of 20-30 min between each trial. Latency to fall down was recorded for each trial and averaged for the 5 trials for each mouse.

### DigiGait

The DigiGait Imaging System (Mouse Specifics, Inc.) was used to assess gait dynamics before crush injury and 7, 14, 21, 28, 35 days post injury. Control and *Dhh^cre/+^;Ninj2^fl/fl^* mice were placed on a motorized treadmill within a plexiglas compartment. Digital video images were acquired at a rate of 80 frames per second by a camera mounted underneath the treadmill to visualize paw contacts on the treadmill belt. The treadmill was set at a fixed speed of 15 cm/s, which was determined as the baseline for both control and *Dhh^cre/+^;Ninj2^fl/fl^* mice. The paw area was calculated by the DigiGait software.

### Sciatic nerve injury

Sciatic nerve injury was performed as previously described 27. The sciatic nerve samples were collected at 0, 7, 14, 21, 28, 35 days post injury.

### GRGDSP administration

GRGDSP (MCE, Cat# HY-P0290A) was first dissolved in DMSO, and then transferred into 0.9% NaCl. Control and *Dhh^cre/+^;Ninj2^fl/fl^* mice were injected with GRGDSP (1 mg/kg) at the injured area daily, from day post injury 7 to 21. In the treatment of Schwann cells, GRGDSP (10 μM) was added to medium for 48 h.

### Experimental design and statistical analysis

For animal experiments, the numbers of mice used in each experiment were indicated in figure legends. For cell experiments, the data were obtained from at least 3 independent experiments. The data for two-group comparisons were analyzed for statistical significance using two-tailed Student's t tests. Error bars represent standard error of measurement (s.e.m.). For multiple comparisons, which were performed using two-way analyses of variance (ANOVAs), the Tukey's multiple-comparison test was used for post-tests. P values are indicated with single asterisk (* P < 0.05), double asterisks (** P < 0.01) and triple asterisks (*** P < 0.001) on graphs. The number for each experiment has been stated in figure legends.

### Data availability

The data that support the findings of this study are available from the corresponding author upon reasonable request.

## Supplementary Material

Supplementary figures.

## Abbreviations

CAMs: cell adhesion molecules
CMAPs: compound muscle action potentials
Co-IP: co-immunoprecipitation assay
ECM: extracellular matrix
FAK: Focal adhesion kinase
GRGDSP: gly-arg-gly-asp-ser-pro integrin-inhibitor which includes RGD-peptide
Gpr126: g-protein coupled receptor 126
ITGB1: integrin subunit beta 1
LN211: laminin 211
Lama2: Laminin Subunit Alpha 2
MS: multiple sclerosis
Ninj2: nerve injury-induced protein 2
NRG1: neuregulin 1
SCs: schwann cells
SNPs: Single nucleotide polymorphisms
TNFα: tumor necrosis factor α
TNFR1: tumor necrosis factor receptor 1
TEM: transmission electron microscope
