# Supplementary material for: Ninj2 regulates Schwann cells development by interfering laminin-integrin signaling
Source: Theranostics. 2022 Oct 17;12(17):7307–18. doi: 10.7150/thno.76131 (PMC9691370; doi:10.7150/thno.76131)
Supplement: Supplementary file 1 — Supplementary figures. [file thnov12p7307s1.pdf]

Ninj2 regulates Schwann cells development by interfering Laminin-integrin signaling

Yuxia Sun[1,5], Xiang Chen[1,5], Cen Yue[2,3,5], Wenyi Yang[1], Shu Zhang[1], Zhimin Ou[4,*], Ying Chen[1,*]

1. State Key Laboratory of Cellular Stress Biology, School of Life Sciences, Xiamen University, Xiamen 361005, Fujian Province, China
2. Department of Neurology, The First Affiliated Hospital of Xiamen University, School of Medicine, Xiamen University, Xiamen, China
3. Fujian Key Laboratory of Brain Tumors Diagnosis and Precision Treatment, Xiamen Key Laboratory of Brain Center, Xiamen, China
4. Women and Children's Hospital, School of Medicine, Xiamen University, Xiamen, China
5.These authors contributed equally to this work.

* Corresponding authors

Correspondence should be addressed to Ying Chen (yingchen@xmu.edu.cn); or Zhimin Ou (ouzm@xmu.edu.cn).

Figure S1

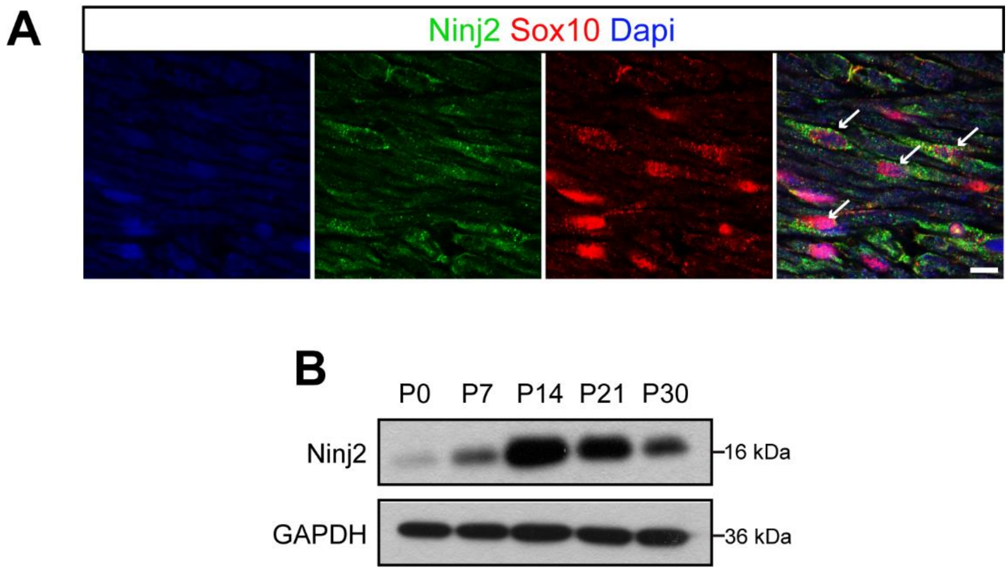

Figure S1. *Ninj2* is dynamically expressed in SCs.

(A) Immunofluorescent staining Ninj2 and Sox10 were performed in the sciatic nerve sections from WT mice at P60. Scale bar, 10 μm. (B) *Ninj2* protein levels were assayed in sciatic nerve of WT mice at the days indicated.

Figure S2

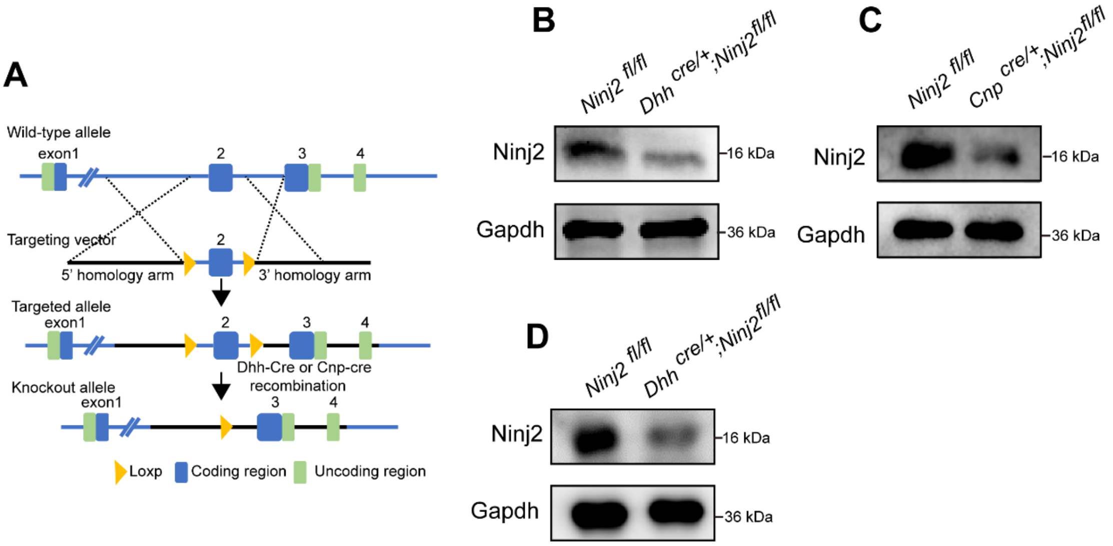

Figure S2. The strategy of generating *Ninj2* conditional knockout mice.

(A) The strategy of generating *Ninj2* conditional knockout mice. (B-D) *Ninj2* protein levels were assayed by western blot in sciatic nerve of WT/$Dhh^{cre/+}$;$Ninj2^{fl/fl}$ mice (B), WT/$Cnp^{cre/+}$;$Ninj2^{fl/fl}$ mice at P60, and primary cultured SCs isolated from WT/$Dhh^{cre/+}$;$Ninj2^{fl/fl}$ mice (D).

Figure S3

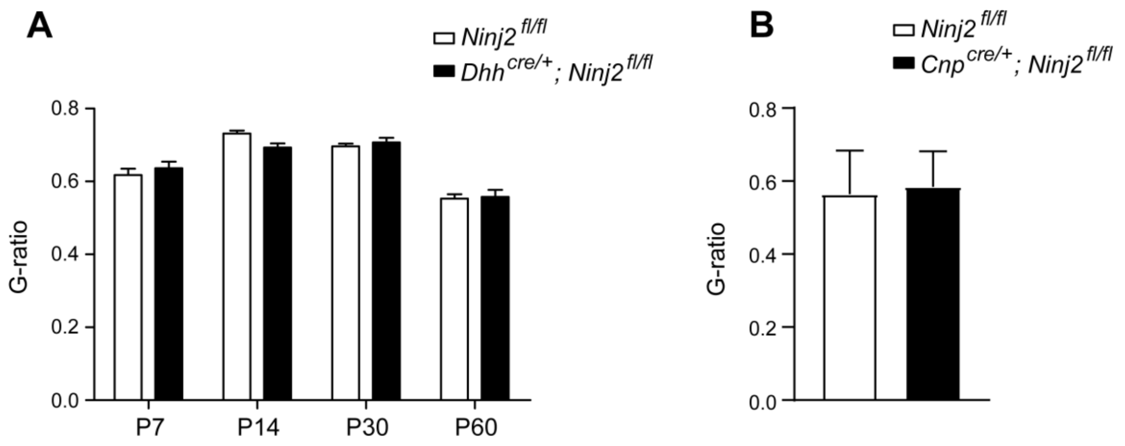

Figure S3. Loss of *Ninj2* has no effect on myelin sheath thickness.

The G-ratio of the sciatic nerve from WT and *Dhh*$^{cre/+}$;*Ninj2*$^{fl/fl}$ mice from postnatal day 7 to 60 as indicated (A), and WT and *Cnp*$^{cre/+}$;*Ninj2*$^{fl/fl}$ mice at P60 (B), were quantified.

Figure S4

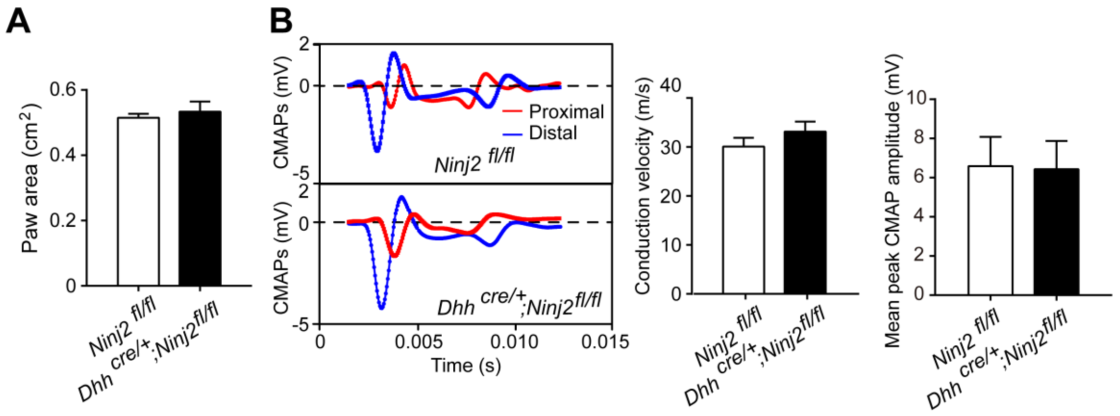

Figure S4. Loss of *Ninj2* has no effect on myelination at adulthood.

(A) Paw area in DigiGait analysis with WT or *Dhh*$^{cre/+}$;*Ninj2*$^{fl/fl}$ mice at P60. (B) Electrophysiological recording of with WT or *Dhh*$^{cre/+}$;*Ninj2*$^{fl/fl}$ mice at P60. For all panels, data were obtained from at least 6 mice.
